# On the clawed lobsters of the genus *Nephropsis* Wood-Mason, 1872 recently collected from deep-sea cruises off Taiwan and the South China Sea (Crustacea, Decapoda, Nephropidae)

**DOI:** 10.3897/zookeys.833.32837

**Published:** 2019-03-25

**Authors:** Su-Ching Chang, Tin-Yam Chan

**Affiliations:** 1 Institute of Marine Biology, National Taiwan Ocean University, Keelung 20224, Taiwan National Taiwan Ocean University Keelung Taiwan; 2 Center of Excellence for the Oceans, National Taiwan Ocean University, Keelung 20224, Taiwan National Taiwan Ocean University Keelung Taiwan

**Keywords:** New records, synonym, taxonomy, West Pacific

## Abstract

Recent deep-sea cruises using Taiwanese research vessels off Taiwan and in the South China Sea yielded seven species of the clawed lobster genus *Nephropsis* Wood-Mason, 1872. Four species are new records for Taiwan (*Nephropsisacanthura* Macpherson, 1990, *N.holthuisi* Macpherson, 1993, *N.serrata* Macpherson, 1993, and *N.suhmi* Bate, 1888) and three species are new records of Dongsha (under the jurisdiction of Taiwan) in the South China Sea (*N.ensirostris* Alcock, 1901, *N.stewarti* Wood-Mason, 1872, and *N.suhmi*). Altogether, five and four species of this genus are now known from Taiwan and Dongsha, respectively. The diagnostic characters and coloration are illustrated for most, if not all, of these species.

## Introduction

Members of the genus *Nephropsis* Wood-Mason, 1872 represent the common clawed lobster found in the deep sea world-wide (Macpherson 1990; Holthuis 1991; Chan 1997; Alves-Júnior et al. 2016). At present, 15 species of this genus are recognized (Chan 2010), and with nine of them distributed in the Indo-West Pacific (Macpherson 1990, 1993; Griffin and Stoddart 1995; Holthuis 1991; Chan 1997; Watabe and Iizuka 1999; Zarenkov 2006). In Taiwanese waters, only one species, *Nephropsisstewarti* Wood-Mason, 1872, has been formally reported (Chan and Yu 1988, 1993), and this species can often been caught by commercial deep-sea trawlers although never in large numbers. Recent deep-sea cruises using Taiwanese research vessels off Taiwan and the South China Sea have yielded many species of *Nephropsis*. Amongst them, *N.serrata* Macpherson, 1993 had been listed in a molecular phylogenetic study (Tshudy et al. 2009). Close examination of this *Nephropsis* material reveals seven species, including four new Taiwanese records (*N.acanthura* Macpherson, 1990, *N.holthuisi* Macpherson, 1993, *N.serrata*, and *N.suhmi* Bate, 1888) and three new records (*N.ensirostris* Alcock, 1901, *N.suhmi*, and *N.stewarti*) around Dongsha (Pratas, under the jurisdiction of Taiwan) in the South China Sea. The present work reports these findings. The two other Indo-West Pacific species those are still not known in Taiwan and adjacent areas are *N.carpenteri* Wood-Mason, 1885, and *N.malhaensis* Borradaile, 1910. Both of them appear to be restricted in the Indian Ocean (Macpherson 1990).

## Materials and methods

Specimens are deposited in the National Taiwan Ocean University, Keelung (**NTOU**). The station (stn) designation is preceded by a prefix indicating the actual type of collecting equipment, as follows: Le Drezen type solo hard bottom 12.4 m otter trawl (CD), 4 m French beam trawl (CP), 2.5 m French beam trawl (PCP), and 3 m ORE beam trawl (OCP). Carapace length (cl) is measured along the dorsal midline from the orbital margin to the posterior margin of the carapace. Morphological terminology mainly follows Macpherson (1990). The synonymy provided is restricted to important taxonomic works of the species and previous Taiwanese and South China Sea records.

## Taxonomy

### Family Nephropidae Dana, 1852

#### Genus *Nephropsis* Wood-Mason, 1872

##### Nephropsis acanthura

Taxon classificationAnimaliaDecapodaNephropidae

Macpherson, 1990

Figs 1A, B , 3

Nephropsis acanthura Macpherson, 1990: 311, figs 5d, 9d–f, 11a, b, 16d (type locality: Philippines); Holthuis 1991: 35, figs 61, 62; Chan 1997: 413; 2010: 156; Poore 2004: 166, fig. 43b; Zarenkov 2006: 85, fig. 3; Poore et al. 2008: 34.

###### Material examined.

TAIWAN 2003, stn CD210, 24°28.99'N, 122°12.79'E, 500–1183 m, 1 Jun 2003, 1 female cl 10.6 mm (NTOU M00951). TAIWAN 2006, stn PCP343, 22°15.699'N, 120°2.131'E, 945–1059 m, 8 Mar 2006, 1 female cl 8.9 mm (NTOU M00952).

**Figure 1.**
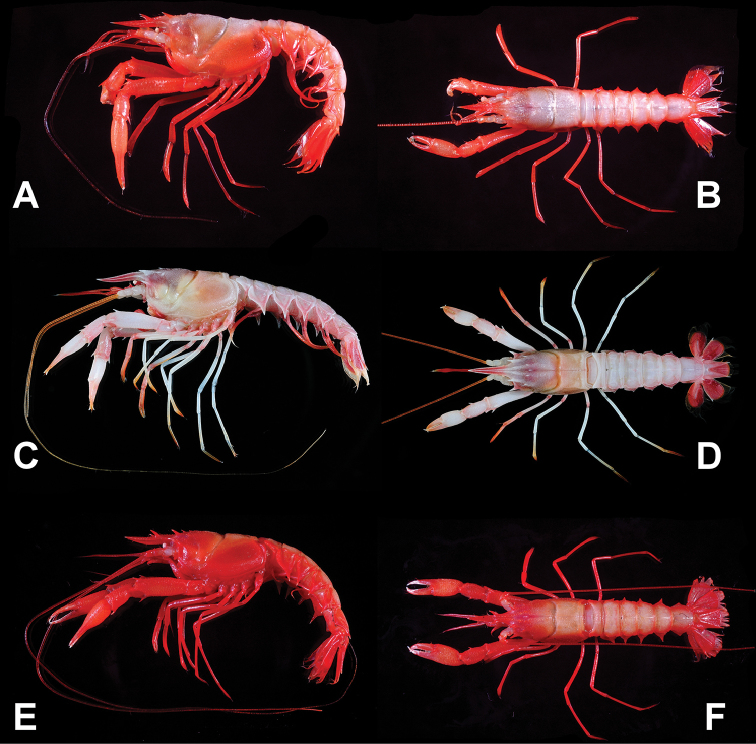
*Nephropsisacanthura* Macpherson, 1990, stn CD210, female cl 10.6 mm (NTOU M00951) **(A, B)**; *N.ensirostris* Alcock, 1901, stn CP4137, female cl 14.6 mm (NTOU M02071) **(C, D)**; *N.holthuisi* Macpherson, 1993, stn CP214, male cl 15.2 mm (NTOU M02160) **(E, F)**.

###### Diagnosis.

Carapace finely granulate. Rostrum longer than half carapace length, bearing a pair of strong lateral spines. Median groove on rostrum extending anteriorly beyond lateral rostral spines. Subdorsal carinae granulate. Supraorbital spines well developed. Postcervical groove passing dorsal midline of carapace. Distance between orbital border and postcervical groove slightly less than twice distance between postcervical groove and posterior border of carapace.

Abdomen with tergites II–VI bearing conspicuous median carina. Anterior border of pleuron II convex and bearing some spinules, terminating in long, acute point. Anterior border of pleura III–V less convex and also terminating in long, acute point. Strong erect dorsal spine present near base of telson. Uropodal exopod with complete diaeresis.

Carpus of cheliped I with strong anterordorsal spine; outer surface without spine; inner border with a spine somewhat at middle of carpus. Carpus of pereiopod II shorter than palm. Carpus of pereiopod III approximately 2/3 palm length. Dactyli of pereiopods IV and V slightly longer than half propodus length.

###### Color in life.

Body generally reddish with dorsal carapace and abdomen whitish. Eyes whitish.

###### Distribution.

Widely distributed in the Indo-West Pacific: Madagascar, Indonesia, Australia, Coral Sea, New Caledonia, Tasman Sea, Chesterfield Islands, the Philippines, southern Japan (Macpherson 1990; Holthuis 1991; Griffin and Stoddart 1995; Chan 1997; Poore 2004; Zarenkov 2006; Poore et al. 2008), and now Taiwan. The bathymetric depth ranges from 500–1305 m.

###### Remarks.

*Nephropsisacanthura* is reported from Taiwan for the first time. This species and *N.occidentalis* Faxon, 1893 are the only two species in the genus bearing an erect spine near the base of the telson (Macpherson 1990; Holthuis 1991). The two Taiwanese specimens fit well with the characteristics of *N.acanthura* in the carapace bearing numerous small granules, the rostrum longer than half carapace length, and the abdominal pleura II-V terminating in a long, acute point (see Macpherson 1990; Holthuis 1991). Nevertheless, there are some variations noticed in the present material compared with those reported by Macpherson (1990). In the Taiwanese specimens, the dactyli of pereiopods IV and V (Fig. 3C) are slightly longer than half propodus length (vs. less than half). The median groove on the rostrum extends beyond the lateral rostral spines (vs. terminating at the level of lateral rostral spines). The distance between the orbital border and postcervical groove is slightly less (vs. slightly more) than twice the distance between the postcervical groove and posterior border of carapace. Additionally, it is reported that the carapace is less granular in the Indonesian material (Chan 1997).

##### Nephropsis ensirostris

Taxon classificationAnimaliaDecapodaNephropidae

Alcock, 1901

Figs 1C, D , 4

**Figure 2.**
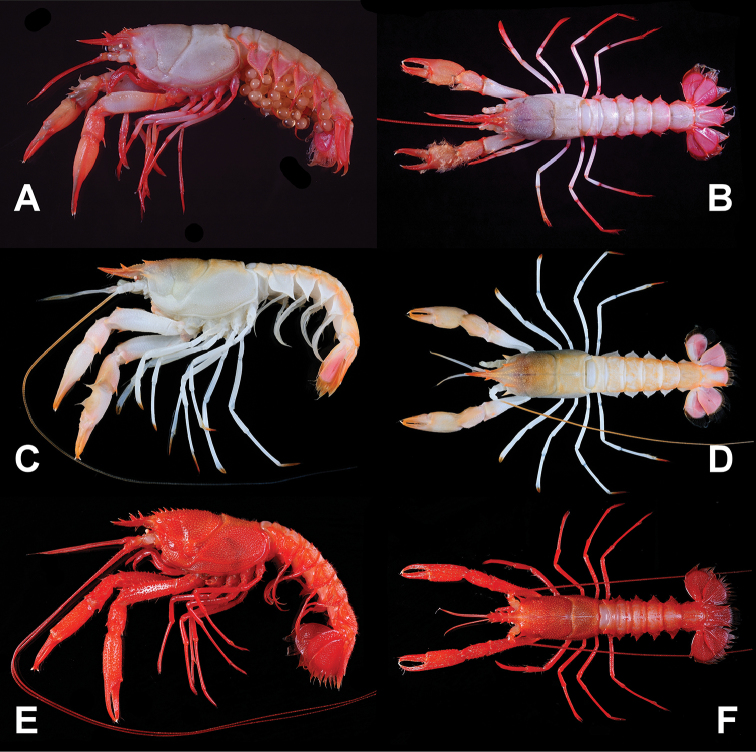
*Nephropsisserrata* Macpherson, 1993 **(A, B)**, stn CP214, ovig. female cl 17.9 mm (NTOU M00157) **(A)**; stn CD210, female cl 17.3 mm (NTOU M02156) **(B)**. *N.stewarti* Wood-Mason, 1872, stn CP4155, female cl 25.2 mm (NTOU M02162) **(C, D)**; *N.suhmi* Bate, 1888, stn OCP280, female 32.4 mm (NTOU M02134) **(E, F)**.

Nephropsis ensirostris Alcock, 1901: 158, pl. 1-fig. 2 (type locality: north of the Laccadives, Arabian Sea); Macpherson 1990: 303, figs 5a, 6, 8a, b, 16a; Holthuis 1991: 41, figs 71, 72; Chan 1997: 414; 2010: 157.

###### Material examined.

Zhongsha 2015, stn CP4137, 19°53.059'N, 114°21.678'E, 536–524 m, 23 Jul 2015, 1 male cl 12.1 mm (NTOU M01831), 1 female cl 14.6 mm (NTOU M02071).

###### Diagnosis.

Carapace finely granulate. Rostrum more than half carapace length, without lateral spine. Median groove reaching or overreaching midpoint of rostrum. Each subdorsal carina with none or two spines and several granules. Gastric tubercle located closer to orbital border than to postcervical groove. Supraorbital and post-supraorbital spines present. Postcervical groove deep, passing dorsal midline of carapace. Pair of dorsal spines located just behind postcervical groove. Distance between orbital border and postcervical groove less than twice distance between postcervical groove and posterior border of carapace.

Abdominal tergites I–V with conspicuous transverse grooves. Dorsal median carina present on tergites II–VI. Anterior borders of pleura II–V granulated, spineless, terminating in a long, acute point. Anterior border of pleuron II more convex than those of other pleura. Uropodal exopod with distinct but incomplete diaeresis.

Cheliped I with little pubescence; carpus with well-developed anterodorsal spine, outer spine on terminal half and inner spine at about mid-length. Carpus of pereiopod II slightly longer than palm. Carpus of pereiopod III more than half palm length. Dactyli of pereiopods IV and V approximately 2/3 propodus length.

###### Color in life.

Body generally pinkish to whitish, with rostrum, tail fan, and antennal and antennular flagella reddish. Eyes whitish.

###### Distribution.

This species has been reported in the Indian Ocean along Gulf of Aden, Laccadive Sea, Bay of Bengal to Andaman Sea. In the western Pacific it is only known from Indonesia and the Philippines. The present material extends its distribution to near Dongsha in the South China Sea. Bathymetric depth ranges from 315 to 1314 m (Macpherson 1990; Holthuis 1991; Chan 1997; Zarenkov 2006).

###### Remarks.

*Nephropsisensirostris* can be readily distinguished from other species of the genus by lacking a lateral spine on the rostrum. The two small specimens collected off west of Dongsha agree well with the description of Macpherson (1990), except for the spines on the subdorsal carina are missing in the male (Fig. 4B; see also Chan 1997). These spines are present in Macpherson’s (1990) material as well as in the female specimen reported here (Figs 1D, 4A).

##### Nephropsis holthuisi

Taxon classificationAnimaliaDecapodaNephropidae

Macpherson, 1993

Figs 1E, F , 5

**Figure 3.**
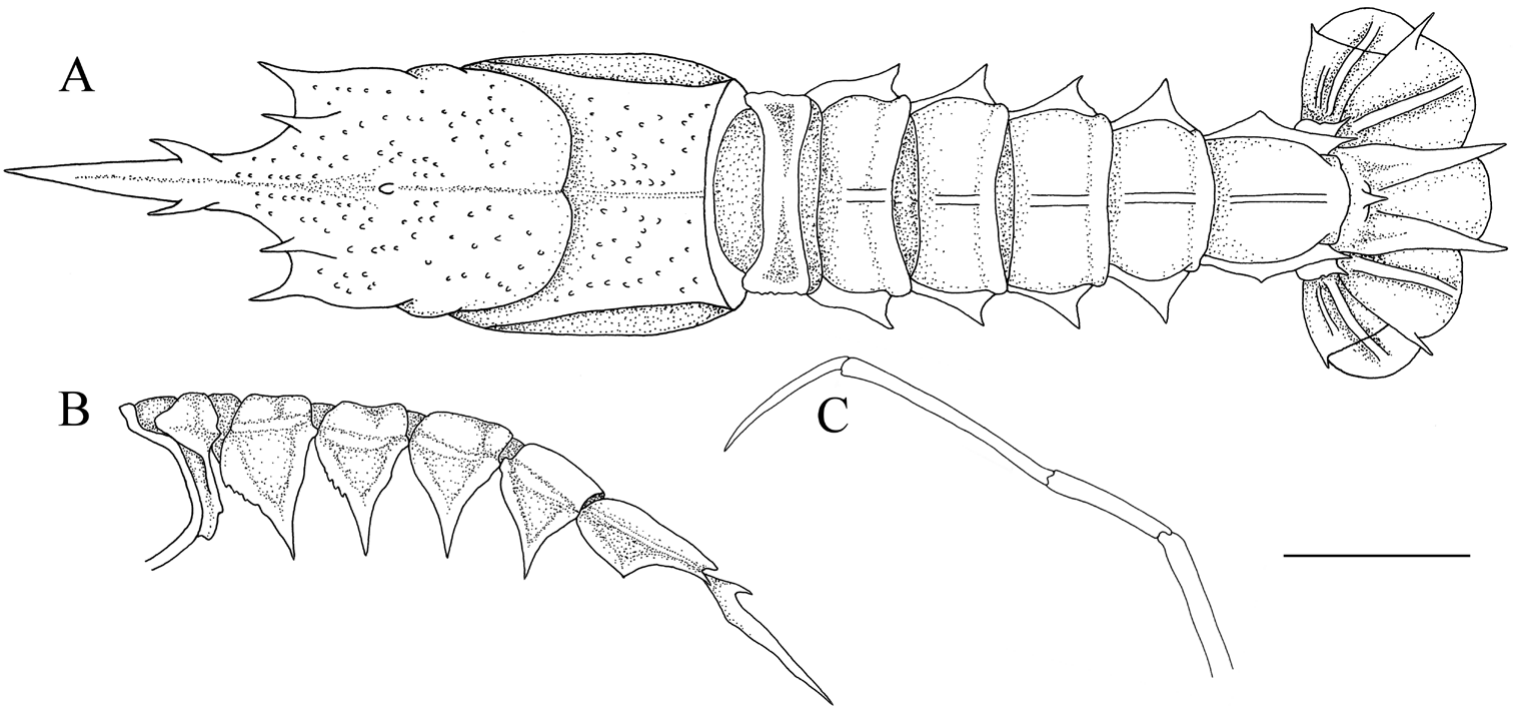
*Nephropsisacanthura* Macpherson, 1990, stnCD 210, female cl 10.6 mm (NTOU M00951). Dorsal habitus **(A)**; abdomen, lateral view **(B)**; left pereiopod V, lateral view **(C)**. Scale bar: 0.5 cm.

Nephropsis holthuisi Macpherson, 1993: 55, figs 1–3 (except fig 3B), fig. 6B (erroneously as N.serrata) (type locality: Ashmore Reef, northwest Australia); Griffin and Stoddart 1995: 234; Chan 1997: 414; 2010: 157; Watabe and Iizuka 1999: 372, figs 1, 2; Poore 2004: 166, fig. 43c.
Nephropsis macphersoni Watabe & Iizuka, 1999: 376, figs 3, 4 (type locality: east of Terrigal, southeastern Australia); Poore 2004: 166, fig. 43d.

###### Material examined.

TAIWAN 2001, stn CD132, 22°20.98'N, 120°6.73'E, 690–700 m, 21 Nov 2001, 1 female cl 18.7 mm (NTOU M02159). TAIWAN 2003, stn CP214, 24°28.59'N, 122°12.66'E, 490–1027 m, 27 Aug 2003, 1 female cl 11.3 mm, 3 males cl 13.6–14.1 mm (NTOU M02158); 1 male cl 15.2 mm (NTOU M02160).

###### Diagnosis.

Carapace sparsely granulate. Rostrum 0.6–0.8 times carapace length, with pair of lateral spines. Median groove on rostrum reaching or overreaching lateral rostral spines. Subdorsal carinae with 1–4 spines posterior to supraorbital spines. Supraorbital spine well-developed, followed by distinct post-supraorbital spine. Distance between level of supraorbital spine and gastric tubercle approximately 0.4 times the distance between gastric tubercle and postcervical groove. Postcervical groove passing dorsal midline of carapace. Distance between orbital border and postcervical groove 1.5–1.9 times distance between postcervical groove and posterior border of carapace.

Abdominal tergites II–VI with distinct dorsal median carina. Anterior border of each pleuron spineless, more convex in pleuron II, and terminating in long, sharp point on pleura II–V. Uropodal exopod with complete diaeresis.

Cheliped I sparsely granulate. Carpus shorter than palm, with anterodorsal spine, a spine on inner dorsal border at midlength, and without any accessory spines or granules. Carpus of pereiopod II somewhat shorter than palm. Carpus of pereiopod III 0.6 times palm length. Dactyli of pereiopods IV and V approximately half propodus length.

###### Color in life.

Body generally vermilion red, with dorsal surface of posterior carapace and abdomen pinkish orange. Tips of large chelae and eyes whitish.

###### Distribution.

Indo-West Pacific: Indonesia, Australia, Japan, and now Taiwan, at depths of 350–1135 m (Macpherson 1993; Griffin and Stoddart 1995; Chan 1997; Watabe and Iizuka 1999; Poore 2004).

###### Remarks.

This species is similar to *Nephropsisrosea* Bate, 1888 from the West Atlantic. They both have one pair of rostral lateral spines, one pair of post-supraorbital spines, a median carina on tergites II–VI, and a complete diaeresis on uropodal exopods. These two species mainly differ in the position of the gastric tubercle (see Macpherson 1993). The Taiwanese material fits the characteristics of *N.holthuisi* in the distance between the supraorbital spine and gastric tubercle being less than half (vs. approx. 2/3 in *N.rosea*) the distance between the gastric tubercle and postcervical groove. Watabe and Iizuka (1999) argued that *N.holthuisi* can be readily distinguished from *N.rosea* by the large spine at the midlength of the inner dorsal border of carpus of cheliped I does not have any accessory spines or granules (vs. 1–3 accessory spines in *N.rosea*). The present specimens also agree in this character.

There are variations in the development of the subdorsal spines in the type series of *N.holthuisi* from rather granulate in the holotype to distinct in the paratype (Macpherson 1993). The present six specimens from Taiwan all have 1–4 distinct spines on the subdorsal carina (Figs 1F, 5). Watabe and Iizuka (1999) considered such difference as specific and treated the paratype of *N.holthuisi* as a distinct species *N.macphersoni* Watabe & Iizuka, 1999. However, all other differences proposed to distinguish *N.macphersoni* from *N.holthuisi* by Watabe and Iizuka (1999), such as pereiopods “less pubescent” or “more robust”, abdominal tergite “more strongly granulated”, are rather vague. Therefore, for the time being *N.macphersoni* is treated as a synonym of *N.holthuisi* as already stated by Chan (2010) until more evidence (e.g., from molecular analysis) is available to support that the former is a distinct species.

##### Nephropsis serrata

Taxon classificationAnimaliaDecapodaNephropidae

Macpherson, 1993

Figs 2A, B , 6

Nephropsis serrata Macpherson, 1993: 59, figs 4–6 (type locality: northwestern Australia); Chan 1997: 414; 2010: 157; Poore 2004: 166, fig. 42b.
Nephropsis hamadai Watabe & Ikeda, 1994: 102, figs 1–2 (type locality: Japan).
Nephropsis lyra Zarenkov, 2006: 87, figs 8–11 (type locality: off northwestern Australia).
Nephropsis pseudoserrata Zarenkov, 2006: 91, figs 15–18 (type locality: northeastern Sumatra).

###### Material examined.

TAIWAN 2003, stn CD210, 24°28.99'N, 122°12.79'E, 500–1183 m, 1 Jun 2003, 3 females cl 9.6–17.3 mm (NTOU M02156); stn CP214, 24°28.59'N, 122°12.66'E, 490–1027 m, 27 Aug 2003, 6 females cl 12.2–23.4 mm, 1 ovig. female cl 18.4 mm, 2 males cl 14.1, 22.5 mm (NTOU M02157); 3 females cl 17.7–20.5 mm, 1 ovig. female cl 17.9 mm (NTOU M00157); 3 females cl 10.7–25.3 mm, 2 ovig. females cl 19.7, 20.1 mm, 5 males cl 9.7–20.7 mm (NTOU M02150). TAIWAN 2006, stn CP371, 24°28.521'N, 122°12.821'E, 582–613 m, 26 Aug 2006, 1 female cl 12.9 mm (NTOU M02151); 1 female cl 13.7 mm, 1 ovig. female cl 17.7 mm, 1 male cl 16.9 mm (NTOU M02154). TAIWAN 2012, stn CP463, 24°28.775'N, 122°12.719'E, 474–647 m, 30 Jun 2012, 2 females cl 11.8, 21.7 mm, 1 ovig. female cl 18.1 mm, 1 male with damaged carapace (NTOU M02152); 1 male cl 10.6 mm (NTOU M02153); 4 females cl 15.6–21.2 mm, 2 ovig. females cl 18.4, 21.7 mm, 3 males cl 13.1–20.3 mm (NTOU M02155).

###### Diagnosis.

Carapace slightly granulate. Rostrum 0.4–0.8 times carapace length, with pair of lateral spines. Median groove reaching lateral rostral spines. Each subdorsal carinae with 2–6 distinct spines and some granules. Supraorbital spine well-developed, without post-supraorbital spine. Postcervical groove passing midline of carapace. Distance between orbital margin and postcervical groove 1.5–1.9 times distance between postcervical groove and posterior margin of carapace.

Abdominal tergites smooth, sometimes with some granules on large specimens, without median dorsal carina. Anterior margins of pleura II–V without spines, usually ending in a long, acute point. Uropodal exopod with complete diaeresis.

Cheliped I sparsely granulated, covered with dense hairs. Carpus with anterodorsal spine, 0–1 spine (rarely 0) on inner dorsal border at midlength, and an anteroventral spine on inner margin. Carpus of pereiopod II more or less as long as palm. Carpus of pereiopod III 0.6 times palm length. Dactyli of pereiopods IV and V 0.5–0.6 times propodus length.

###### Color in life.

Body generally whitish with rostrum, distal parts of pereiopods, maxilliped III, antennular and antennal flagella, abdominal pleura, uropods and distal part of telson pinkish red to reddish. Eyes whitish. Eggs greyish yellow.

###### Distribution.

Recorded from Indonesia, Australia, Japan, and now Taiwan, at depths of 390–1430 m (Macpherson 1993; Watabe and Ikeda 1994; Chan 1997; Poore 2004; Zarenkov 2006).

###### Remarks.

One of the specimens from the lot NTOU M00157 was used and listed in a recent molecular phylogenetic work (Tshudy et al. 2009: table 1), which is the first literature record of this species from Taiwan. *Nephropsisserrata* is very similar to *N.stewarti*. They both lack median dorsal carina on the abdomen and mainly differ in the presence or absence of spines on the subdorsal carina (Macpherson 1993). The other distinguishing characters mentioned by Macpherson (1993), such as *N.serrata* having more elongate lateral rostral spines than supraorbital spines, a slightly shorter rostrum, and a less elongate large chela, are difficult to use (see Macpherson 1993: Figs 7, 8). The Taiwanese specimens all bear distinct spines on the subdorsal carinae and agree well with the original description of the species (Macpherson 1993), except for the carpus of the large cheliped bearing 0–1 (mostly one) rather than two spines on the inner dorsal border at mid-length. Moreover, an ovigerous female (NTOU M02154) is abnormal in having two spines on the right side of the rostrum.

Three recently described species, namely *N.hamadai* Watabe & Ikeda, 1994, *N.lyra* Zarenkov, 2006, and *N.pseudoserrata* Zarenkov, 2006, are treated under the synonyms of *N.serrata* by Chan (1997, 2010). Some of the differences between *N.hamadai* and *N.serrata* proposed by Watabe and Ikeda (1994) have been shown to be inappropriate by Chan (1997). The present Taiwanese material also reflects such an opinion, except for the inner dorsal border of the carpus of the large cheliped always being armed with fewer than two spines at the mid-length (two spines in *N serrata* by Macpherson 1993 and one spine in *N.hamadai* by Watabe and Ikeda 1994). In the original description of *N.lyra*, Zarenkov (2006) argued that this species is closest to *N.stewarti* and *N.grandis*Zarenkov 2006. However, *N.lyra* is actually most similar to *N.serrata* in bearing 3–4 distinct spines on the subdorsal carina. Since no distinct difference is observed between the original illustrations of *N.lyra* (Zarenkov 2006: figs 8–11) from *N.serrata*, and the type localities of both species are from the same area (i.e., off northwestern Australia), these two species are considered as synonyms pending more evidence to support their separation. Another species, *N.pseudoserrata* described by Zarenkov (2006), based on a single specimen from Sumatra, is also closest to *N.serrata* in having 1–2 spines on the subdorsal carina. However, Zarenkov (2006) claimed that *N.serrata* differs from *N.pseudoserrata* in the subdorsal carina being smooth. As this separation is based on a misinterpretation and the other differences proposed by Zarenkov (2006: table 4) on the armature of the large cheliped are rather variable in this genus, *N.pseudoserrata* is not recognized as a species distinct from *N.serrata*.

**Figure 4.**
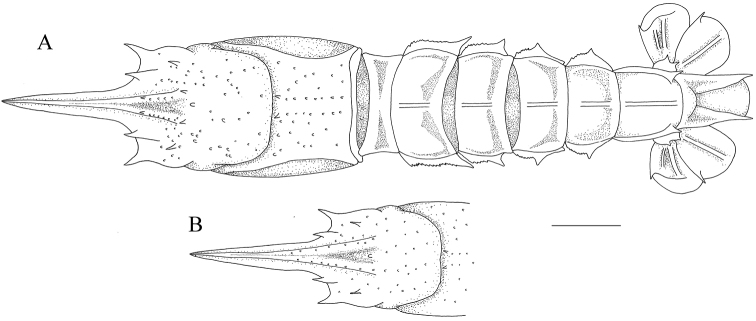
*Nephropsisensirostris* Alcock, 1901, stn CP4137. Female cl 14.6 mm (NTOU M02071), dorsal habitus **(A)**; male cl 12.1 mm (NTOU M01831), anterior carapace, dorsal view **(B)**. Scale bar: 0.5 cm.

##### Nephropsis stewarti

Taxon classificationAnimaliaDecapodaNephropidae

Wood-Mason, 1872

Figs 2C, D , 7

Nephropsis stewarti Wood-Mason, 1872: 60 (type locality: Ross Island, Andaman Sea); Chan and Yu 1988: 8, pl. 1A; 1993: 83, unnumbered photo; Macpherson 1990: 312, figs 5e, 10, 11c, d, 16e; 1993: 63; Holthuis 1991: 45, figs 80, 81; Chan 1997: 415; 2010: 157; Zarenkov 2006: 93, fig. 19; Poore et al. 2008: 34.
Nephropsis grandis Zarenkov, 2006: 86, figs 5–7 (type locality: off Arnhem Land, northern Australia).

###### Material examined.

Zhongsha 2015, stn CP4137, 19°53.059'N, 114°21.678'E, 536–524 m, 23 Jul 2015, 1 male cl 15.9 mm (NTOU M02161); stn CP4155, 16°13.60'N, 115°01.61'E, 526–510 m, 28 Jul 2015, 1 female cl 25.2 mm (NTOU M02162), 1 male cl 12.8 mm (NTOU M02163). Yilan County, Dasi fishing port, 10 Sept 1984, 1 female cl 44.8 mm (NTOU M02165); Sept 1992, 2 females cl 39.7, 39.8 mm, 1 male cl 45.3 mm (NTOU M02171); Aug 2003, 1 male cl 38.9 mm (NTOU M00505); 29 May 2008, 1 male cl 41.9 mm (NTOU M02177); 12 Apr 2012, 1 female cl 32.4 mm (NTOU M02178); 14 Aug 2013, 1 male cl 19.8 mm (NTOU M02179). Yilan County, Nanfang-ao fishing port, 2 May 1985, 1 female cl 40.7 mm, 1 male with damaged carapace (NTOU M02166); 20 Apr 1988, 1 female cl 40.5 mm, 1 male cl 28.2 mm (NTOU M02167); 12 Nov 2004, 1 male cl 31.8 mm (NTOU M02176). Pingtung County, Donggang fishing port, Jul 1975, 1 male cl 23.4 mm (NTOU M02164); 3 Mar 1991, 6 females cl 19.5–20.2 mm, 1 male cl 21.1 mm (NTOU M02168); 14 May 1991, 2 males cl 19.2, 21.7 mm (NTOU M02169); 4 Jun 1995, 1 male cl 22.4 mm (NTOU M02173); 27 Dec 1997, 2 males cl 29.0, 32.7 mm (NTOU M02174), 1 male cl 21.8 mm (NTOU M02175); 2 Oct 2014, 1 male cl 25.6 mm (NTOU M01898). Taiwan, locality not specified, 1993, 2 females cl 30.2, 40.6 mm, 1 male cl 31.1 mm (NTOU M02172). Dongsha, Jun 1991, 1 female cl 45.9 mm (NTOU M02170).

**Figure 5.**
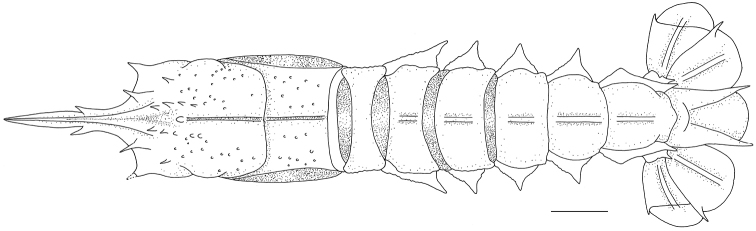
*Nephropsisholthuisi* Macpherson, 1993, stn CP132, female cl 18.7 mm (NTOU M02159), dorsal habitus. Scale bar: 0.5 cm.

###### Diagnosis.

Carapace nearly smooth, sometimes with some granules. Rostrum with pair of lateral spines. Median groove overreaching lateral rostral spines. Subdorsal carinae granulate, without spines. Supraorbital spines well developed, without post-supraorbital spine. Distance between orbital margin and postcervical groove more than 1.5 times distance between postcervical groove and posterior margin of carapace.

Abdominal tergites II–V without dorsal median carina. No spines on anterior margin of each pleuron. Anterior margin of pleuron II convex, generally ending in long, sharp point (but rather short and blunt in large specimens). Anterior margins of pleura III–V less convex, each ending in long, sharp point. Uropodal exopod with distinct and complete diaeresis.

Cheliped I densely pubescent. Carpus with anterodorsal and anteroventral spines, and 0–4 dorsal spines on outer margin. Carpus of pereiopod II slightly shorter than palm. Carpus of pereiopod III more than half palm length. Dactyli of pereiopods IV and V approximately half propodus length.

###### Color in life.

Body generally whitish with antennular and antennal flagella, anterior segment of large chelae, dorsal carapace, and abdomen somewhat pale orange. Rostrum, tips of pereiopods II to V, and tail fan pinkish red. Eyes whitish. Pubescence on body light grey and eggs whitish.

###### Distribution.

Widely distributed in the Indo-West Pacific and has been reported from Madagascar, Natal, Mozambique, Kenya, Gulf of Aden, Andaman Sea, Bay of Bengal, Indonesia, Australia, the Philippines, Japan, Taiwan, and now the South China Sea, at depths of 170 to more than 1060 m (Macpherson 1990, 1993; Chan 1997; Zarenkov 2006; Poore et al. 2008).

**Figure 6.**
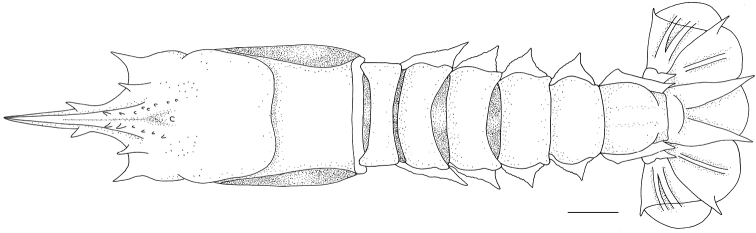
*Nephropsisserrata* Macpherson, 1993, stn CP463, female cl 21.7 mm (NTOU M02152), dorsal habitus. Scale bar: 0.5 cm.

###### Remarks.

The present material collected by Taiwanese research vessels were from the South China Sea; near Dongsha (NTOU M02161, M02170) or the center of the South China Sea (NTOU M02162, M02163).

*Nephropsisgrandis* Zarenkov, 2006 was described based on a single specimen from northern Australia, and is extremely similar to *N.stewarti* in the subdorsal carina lacking a spine and the abdomen without any dorsal median carina. These two species were differentiated only by the carpus of the large cheliped, which is more spiny in *N.grandis* (cf. Zarenkov 2006: table 4). As there are generally large intraspecific variations in the spination on the large cheliped in *Nephropsis* (e.g., in the present abundant material there are 4–15 distinct spines on the carpus of the large cheliped), more comprehensive studies with molecular genetic comparisons are necessary to verify if large cheliped spination is indeed a good character in separating the species of this genus. Therefore, for the time being *N.grandis* is treated as a synonym of *N.stewarti* as stated by Chan (2010).

##### Nephropsis suhmi

Taxon classificationAnimaliaDecapodaNephropidae

Bate, 1888

Figs 2E, F , 8

Nephropsis suhmi Bate, 1888: 181, pl. 23-fig. 3, pl. 24-fig. 2 (type locality: Aru Islands, Indonesia); Macpherson 1990: 306, figs 5b, 7d-f, 8c, d, 16b; 1993: 64; Holthuis 1991: 46, figs 60b, 82; Griffin and Stoddart 1995: 234; Poore 2004: 166, fig. 43e; Zarenkov 2006: 93; Chan 2010: 157; Yaldwyn and Webber 2011: 198.
Nephropsis meteor Zarenkov, 2006: 90, figs 12–14 (type locality: Gulf of Aden).

###### Material examined.

TAIWAN 2002, stn CP189, 21°39.91'N, 118°20.94'E, 1649–1629 m, 27 Aug 2002, 1 female cl 26.6 mm (NTOU M02131). TAIWAN 2004, stn CD238, 25°12.28'N, 123°1.85'E, 1689–1650 m, 23 Jul 2004, 1 male cl 17.8 mm (NTOU M02132). TAIWAN 2005, stn CP278, 24°23.63'N, 122°14.13'E, 1222–1239 m, 14 Jun 2005, 1 female cl 17.9 mm (NTOU M02133); stn OCP280, 24°23.71'N, 122°14.22'E, 1213–1261 m, 14 Jun 2005, 1 female 32.4 mm, 1 male cl 28.1 mm (NTOU M02134). TAIWAN 2006, stn CP372, 24°23.619'N, 122°14.138'E, 1220–1280 m, 26 Aug 2006, 1 female cl 12.9 mm (NTOU M02135). NanHai 2014, stn CP4106, 10°19.1500'N, 114°14.2530'E, 1292–1321 m, 6 Jan 2014, 1 female cl 18.5 mm (NTOU M02136); stn CP4108, 10°23.3701'N, 114°23.2672'E, 1707–1799 m, 6 Jan 2014, 1 female cl 35.7 mm (NTOU M02137), 1 male cl 38.3 mm (NTOU M02138), 1 male cl 34.8 mm (NTOU M02139). Dongsha 2014, stn CP4122, 21°34.976'N, 118°14.2792'E, 1713–1624 m, 30 Apr 2014, 1 female cl 40.4 mm (NTOU M02140). — Zhongsha 2015, stn CP4134, 19°55.837'N, 116°25.368'E, 1128–1278 m, 22 Jul 2015, 1 female cl 16.8 mm, 1 male cl 16.7 mm (NTOU M02141); stn CP4141, 18°54.31'N, 113°58.27'E, 1151–1286 m, 24 Jul 2015, 1 female cl 16.1 mm (NTOU M02142); stn CP4157, 19°52.593'N, 116°27.145'E, 1205–1389 m, 29 Jul 2015, 1 female cl 28.8 mm, 2 males cl 12.7, 16.0 mm (NTOU M02143); stn CP4163, 21°38.534'N, 118°19.179'E, 1683–1643 m, 31 Jul 2015, 1 female cl 46.8 mm (NTOU M02144); 1 female cl 39.4 mm, 1 male cl 19.9 mm (NTOU M02145); stn CP4167, 22°6.125'N, 119°7.775'E, 1756–1306 m, 1 Aug 2015, 1 male cl 13.2 mm (NTOU M02146). Cold Seep Cruise 2016, stn CST 11, 22°8.830'N, 119°15.681'E, 1319–1176 m, 27 Apr 2016, 1 female with damaged carapace (NTOU M02147); stn CST 17, 22°3.791'N, 118°58.804'E, 1483 m, 1 May 2016, 1 male cl 16.0 mm (NTOU M02148). Dongsha, 1256 m, 25 Apr 1996, 1 male cl 23.1mm (NTOU M02149).

**Figure 7.**
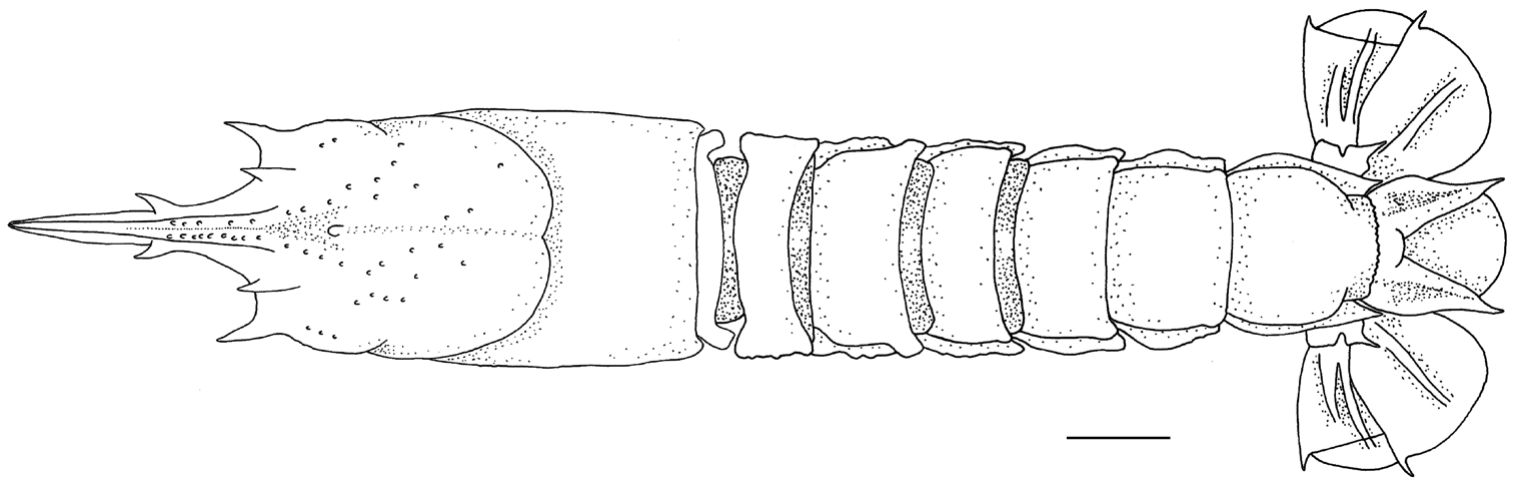
*Nephropsisstewarti* Wood-Mason, 1872, Dasi fishing port, Yilan County, male cl 38.9 mm (NTOU M00505), dorsal habitus. Scale bar: 1 cm.

###### Diagnosis.

Carapace covered with numerous granules of varying sizes (more developed in adults). Rostrum 0.4–0.6 times carapace length (somewhat longer in smaller specimens), bearing two (rarely three) lateral spines on each side, sometimes with one additional spine. Median groove reaching or almost reaching distal pair of lateral rostral spines. Each subdorsal carina with 0–7 (usually 3–5) spines and some granules. Gastric tubercle closer to supraorbital spine than to postcervical groove. Supraorbital spine well developed. Post-supraorbital spine present, usually followed by 1–2 spines. Postcervical groove deep, crossing dorsal midline. Distance between orbital border and postcervical groove 1.5–1.9 times distance between postcervical groove and posterior border of carapace.

Abdomen covered with granules. Tergites I–V each with distinct transverse groove interrupted medially. Pleura II–V slightly convex, each terminating in long, acute point which occasionally absent on pleuron V. Anterior border of pleura II and III usually bearing one strong spine (sometimes two) and some additional spinules. Anterior border of pleuron IV sometimes with a spine as well. Posterobasal border of pleuron V usually unarmed but occasionally bearing a single large spine. Dorsal surface of tail fan granulate; uropodal exopod lacking diaeresis.

Cheliped I bearing numerous granules; carpus with well-developed anterodorsal spine; outer surface bearing several spines (sometimes only 1–2 distinct spines in smaller specimens); inner surface with anteroventral spine and 1–2 (rarely 0) spines medially; dorsal surface of merus lined with spines. Carpus of pereiopod II 0.6–0.9 times palm length. Carpus of pereiopod III slightly more than half palm length. Dactyli of pereiopods IV and V approximately half propodus length.

###### Color in life.

Entire body vermilion red, except tips of large chelae, eyes, most dorsal parts of abdominal tergites I to V, and basal parts of antennular peduncles whitish.

###### Distribution.

Widely distributed in the Indo-West Pacific and recorded from Madagascar, Gulf of Aden, Maldive Sea, Arabian Sea, Indonesia, Australia, New Caledonia, western Tasman Sea, New Zealand, at 786–2029 m deep (Macpherson 1990, 1993; Griffin and Stoddart 1995; Poore 2004; Zarenkov 2006; Yaldwyn and Webber 2011). This species is reported for the first time from Taiwan and the South China Sea (including Dongsha).

**Figure 8.**
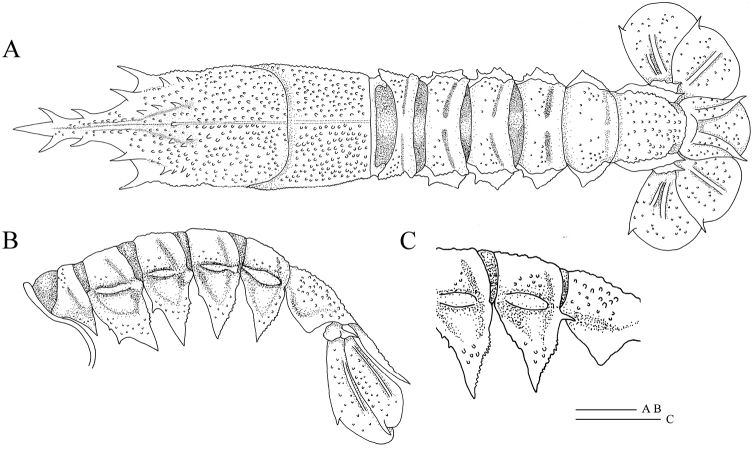
*Nephropsissuhmi* Bate, 1888, stn CP4163. Female cl 39.4 mm (NTOU M02145) **(A, B)**; stn CP4108, female cl 38.3 mm (NTOU M02138) **(C)**. Dorsal habitus **(A)**; abdomen, lateral view **(B)**; abdomen somite V, lateral view **(C)**. Scale bar: 1 cm.

###### Remarks.

*N.suhmi* from the Indo-West Pacific and *N.agassizii* A. Milne-Edwards, 1880 from the West Atlantic (Macpherson 1990; Alves-Júnior et al. 2016) are the only two known species of *Nephropsis* lacking a diaeresis on uropodal exopods. The present material fits well with the concept of *N.suhmi* in having the dactylus of pereiopod V approximately half the propodus length (vs. distinctly less than half in *N.agassizii*; Holthuis 1974: fig. 19; Macpherson 1990: fig. 7c; Alves-Júnior et al. 2016: fig. 1A). The only discrepancy may be that in two (female of NTOU M02134; NTOU M02138) of the present 24 specimens there is a large posterior spine at the base of the pleuron V (Fig. 8C).

*Nephropsismeteor* Zarenkov, 2006 is closely related to *N.suhmi* and was described based on a single specimen from the Gulf of Aden (Zarenkov 2006). The characters separating *N.meteor* from *N.suhmi* are the merus of large cheliped with two instead of one rows of spines, the postcervical groove dorsally armed with a pair of dorsal spines (vs. no dorsal spines), and the anterior margin of abdominal pleura III–V each bearing two spines instead of one spine (Zarenkov 2006: table 3). However, in the original description and illustration of *N.meteor* (Zarenkov 2006: 90, fig. 13B), the anterior margins of pleura IV and V each bearing only one and not two spines as listed in the table of distinguishing characters given by Zarenkov (2006: table 3). In the present material, there are 0–2 distinct spines on the anterior margin of each of the abdominal pleura III–V. The spination on the postcervical groove and merus of large cheliped are also rather variable in the abundant material examined in this study (i.e., 1–2 rows of spines on the merus of the large cheliped and 0–2 distinct spines on the dorsal part of postcervical groove). Thus, *N.meteor* should be considered as a synonym of *N.suhmi* as stated by Chan (2010) until there is more evidence to support their separation.

##### Nephropsis sulcata

Taxon classificationAnimaliaDecapodaNephropidae

Macpherson, 1990

Fig. 9

Nephropsis sulcata Macpherson, 1990: 319, figs 13e–g, 14a, b, 15a, b, 16g (type locality: Philippines); 1993: 64; Holthuis 1991: 47, figs 84, 85; Griffin and Stoddart 1995: 235, fig. 1; Chan 1997: 415; 2010: 157; Zarenkov 2006: 94, fig. 20A.
Nephropsis atlantica : Bruce 1966: 223. **non** Norman, 1882

###### Material examined.

Dongsha 2014, stn CP4130, 20°17.971'N, 116°07.966'E, 795–822 m, 2 May 2014, 1 male cl 16.6 mm (NTOU M02130).

###### Diagnosis.

Carapace generally smooth, with some small granules. Rostrum more than half carapace length, bearing two strong lateral spines. Median groove overreaching distal pair of lateral rostral spines. Posterior portion of subdorsal carina armed with several small spines. Postorbital and post-supraorbital spines present. Postcervical groove crossing midline of carapace. Distance between orbital margin and postercervical groove approximately 1.5 times distance between postcervical groove and posterior margin of carapace.

Abdominal tergites II–VI with distinct median carina. Anterior border of pleura II–V convex, each terminating in long, acute point. A single strong spine and 1–3 additional spines on anterior border of pleuron II. Anterior border of pleuron III with two small spines. Posterior border of pleuron V armed with a strong spine. Uropodal exopod with complete diaeresis.

Cheliped I bearing numerous granules on all articles. Carpus with anterodorsal and anteroventral spines; two spines on inner surface; outer surface with one spine on distal half. Carpus of pereiopod III 0.79 times palm length.

**Figure 9.**
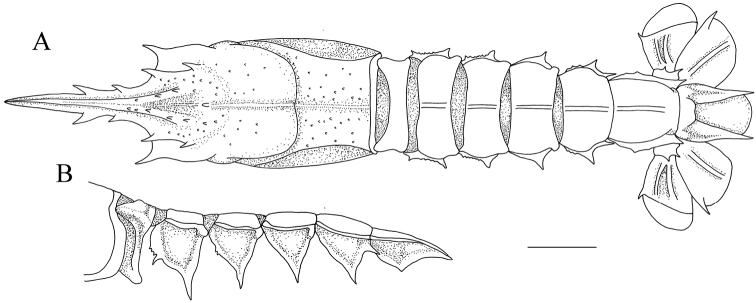
*Nephropsissulcata* Macpherson, 1990, stn CP4130, male cl 16.6 mm (NTOU M02130). Dorsal habitus **(A)**; abdomen, lateral view **(B)**. Scale bar: 0.5 cm.

###### Color in life.

Unknown.

###### Distribution.

Widely distributed in the Indo-West Pacific from Madagascar, Laccadive Sea, Indonesia, South China Sea, northwestern and eastern Australia, Coral Sea, Chesterfield Islands, New Caledonia, and the Philippines, at depths of 200–1115 m (Macpherson 1990, 1993; Holthuis 1991; Griffin and Stoddart 1995; Chan 1997; Zarenkov 2006).

###### Remarks.

The present specimen was collected off Dongsha in the South China Sea and the presence of *N.sulcata* off Dongsha has been reported before (Bruce 1966, as *N.atlantica* Norman, 1882; see Macpherson 1990). *Nephropsissulcata* can be distinguished from the closely related Atlantic species *N.atlantica* by the carpus of the pereiopod II being longer than the palm, and usually bearing a spine on the posterior border of the abdominal pleuron V (Fig. 9B; see also Chan 1997). Even though both sides of pereiopod II are missing in the present specimen, its remaining part agrees well with the description of *N.sulcata* by Macpherson (1990).

#### Key to species of *Nephropsis* off Taiwan and Dongsha

**Table:** 

1	Rostrum without lateral spines	*** N. ensirostris ***
–	Rostrum with lateral spines	**2**
2	Exopod of uropod without diaeresis	*** N. suhmi ***
–	Exopod of uropod with diaeresis	**3**
3	Rostrum with 2 pairs of lateral spines	*** N. sulcata ***
–	Rostrum with 1 pair of lateral spines	**4**
4	Dorsal surface of telson with well-developed proximal median spine	*** N. acanthura ***
–	Dorsal surface of telson without median spine	**5**
5	Abdominal tergites with dorsal median carina	*** N. holthuisi ***
–	Abdominal tergites without dorsal median carina	**6**
6	Subdorsal carinae of rostrum with distinct spines	*** N. serrata ***
–	Subdorsal carinae of rostrum without distinct spines	*** N. stewarti ***

## Supplementary Material

XML Treatment for Nephropsis acanthura

XML Treatment for Nephropsis ensirostris

XML Treatment for Nephropsis holthuisi

XML Treatment for Nephropsis serrata

XML Treatment for Nephropsis stewarti

XML Treatment for Nephropsis suhmi

XML Treatment for Nephropsis sulcata
